# High-Throughput RNA FISH Analysis by Imaging Flow Cytometry Reveals That Pioneer Factor Foxa1 Reduces Transcriptional Stochasticity

**DOI:** 10.1371/journal.pone.0076043

**Published:** 2013-09-20

**Authors:** Avin S. Lalmansingh, Kamalpreet Arora, Richard A. DeMarco, Gordon L. Hager, Akhilesh K. Nagaich

**Affiliations:** 1 Office of Biotechnology Products, Center for Drug Evaluation and Research, Food and Drug Administration, Bethesda, Maryland, United States of America; 2 Amnis, EMD Millipore Corporation, Seattle, Washington, United States of America; 3 Laboratory of Receptor Biology and Gene Expression, National Cancer Institute, National Institute of Health, Bethesda, Maryland, United States of America; Baylor College of Medicine, United States of America

## Abstract

Genes are regulated at the single-cell level. Here, we performed RNA FISH of thousands of cells by flow cytometry (flow-RNA FISH) to gain insight into transcriptional variability between individual cells. These experiments utilized the murine adenocarcinoma 3134 cell line with 200 copies of the MMTV-Ras reporter integrated at a single genomic locus. The MMTV array contains approximately 800–1200 binding sites for the glucocorticoid receptor (GR) and 600 binding sites for the pioneer factor Foxa1. Hormone activation of endogenous GR by dexamethasone treatment resulted in highly variable changes in the RNA FISH intensity (25–300 pixel intensity units) and size (1.25–15 µm), indicative of probabilistic or stochastic mechanisms governing GR and cofactor activation of the MMTV promoter. Exogenous expression of the pioneer factor Foxa1 increased the FISH signal intensity and size as expected for a chromatin remodeler that enhances transcriptional competence through increased chromatin accessibility. In addition, specific analysis of Foxa1-enriched cell sub-populations showed that low and high Foxa1 levels substantially lowered the cell-to-cell variability in the FISH intensity as determined by a noise calculation termed the % coefficient of variation. These results suggest that an additional function of the pioneer factor Foxa1 may be to decrease transcriptional noise.

## Introduction

The control of gene transcription is increasingly being recognized as a probabilistic or stochastic process that requires analyses at the single cell level to precisely define the underlying mechanisms [1], [2]. The use of sensitive techniques such as fluorescence *in situ* hybridization (FISH) analysis of nascent mRNA transcripts is ideally suited to measure cell-to-cell variability associated with stochastic gene expression. Regulated genes tend to show large variability in expression between individual cells, resulting in an asymmetric or skewed distribution curve for a given cell population [3], [4]. By contrast, genes expressed at a steady-state show lower variability which result in a Poisson distribution curve as in the case of yeast housekeeping genes that are constitutively active [5], [6]. The increased transcriptional variability associated with regulated genes compared to that of constitutively active genes is predicted to be due to infrequent but intense episodes or ‘bursts’ of transcriptional activity [6], [7]. Furthermore, the ‘bursting’ response is suggested to be caused by random chromatin remodeling as gene promoters transition between inaccessible and accessible states.

The packaging of DNA into higher-order chromatin structure represents a major barrier to regulatory factors that bind to their target DNA sites in chromatin to control transcription. Chromatin accessibility is controlled by forkhead box (FOX) proteins that function as pioneer factors in chromatin to loosen up the tightly packaged nucleosomal DNA for more favorable regulatory factor binding [8]. This special property of pioneer factors to enable other factors to load onto chromatin results in enhanced transcriptional competence of target genes [9]. Some early insights into the mechanism of pioneering function were predicted from structural studies of FOXA3. The crystal structure of the FOXA3 DNA binding domain (DBD) revealed that it folds into a helix-turn-helix motif with adjacent polypeptide loops or ‘wings’ resembling the winged-helix structure of linker histone H5 [10]. However, despite the structural similarity, both FOXA family of proteins and linker histones remain functionally divergent. Linker histones promote chromatin compaction whereas pioneer factors open up chromatin to enhance accessibility. The unique pioneering function of FOX proteins emanates from bimodal interactions with chromatin. The central DBD region of Foxa1 provides DNA sequence-specific binding, whereas transcriptional activator regions present within the Foxa1 C-terminus interact with core histones H3 and H4 to promote chromatin opening [11], [12]. Recent live cell imaging studies indicate a role for the C-terminus to increase Foxa1 mobility within the nucleoplasm relative to linker histone H1 [13]. Although these protein dynamics studies suggest that Foxa1 competition with linker histones contributes to its pioneering function, linker histone antagonism may also influence transcriptional variability in individual cells.

To gain insight into how the Foxa1 pioneering function influences transcriptional stochasticity, we carried out studies using the hormone-inducible mouse mammary tumor virus (MMTV) gene. The MMTV gene harbors three Foxa1 DNA binding sites in close proximity to six glucocorticoid receptor (GR) binding sites within the 400 base pair region of the proximal promoter [14]. We utilized the murine 3134 adenocarcinoma cell line engineered with 200 tandem repeats of the MMTV transgene driving Harvey viral Ras stably integrated at a single locus in the cell’s genome [15], [16]. This multi-copy MMTV transgene (also called the MMTV array) is estimated to have 800–1200 GR DNA binding sites and 600 Foxa1 DNA binding sites altogether. Previous FISH studies using several 3134 cell line subclones expressing fluorescently-tagged proteins demonstrated probabilistic GR and coregulator interactions with the MMTV array that contributed to variable transcriptional responses within randomly sampled cells [17], [18]. In the current study we adapted an RNA FISH procedure for use in fluid suspension that allowed downstream analysis of transcriptional variability in thousands of cells by flow-RNA FISH using the ImageStream imaging flow cytometry platform. This technology enabled quantification of the FISH signal intensity and size as readouts of transcription, and also provided spatial resolution of Foxa1 nuclear localization at the FISH foci. We show that exogenous Foxa1 expression increases the average amount of nascent transcripts produced per active MMTV array and at the same time decreases the transcriptional variability between cells. Taken together, these results indicate that Foxa1 reduces noise and promotes a more uniform transcriptional response.

## Materials and Methods

### FISH DNA probe generation

A Digoxigenin (DIG)-labeled DNA probe was prepared by nick-translation of the pM18 plasmid that contains the viral-Ha-Ras coding sequence [19], [20]. Nick-translation was carried out according to instructions provided with the DIG-Nick translation kit (Roche).

### RNA FISH in a cell suspension

Cells were seeded on 100 mm dishes and maintained in phenol-red free DMEM containing 5% charcoal-stripped serum. The following day, cells were transfected with 5 µg control pcDNA3.1 or V5 tagged-Foxa1 per 3×10^6^ cells using Lipofectamine 2000 (Invitrogen). At 24 hrs post transfection, hormone-deprived cells were treated with vehicle (0.1% ethanol) or 100 nM dexamethasone (dex) for 1 hr. Time course experiments were performed with non-transfected cells that were collected for processing of the FISH signals at 5, 30, 60, 120 and 240 mins after 100 nM dex treatment. Cells were harvested by trypsinization and processed for RNA FISH and immunostaining in cell suspension for detection and quantification using the ImageStream imaging flow cytometry technology as follows.

Trypsin-detached cells were pelleted at 900 *x g* for 10 mins at 4 °C, washed with 1X PBS and transferred to 1.5 mL eppendorf tubes to carry out the FISH/immunostaining protocol. Cells were fixed with 4% paraformaldehyde in PBS (1000 µl) for 20 mins at room temperature, pelleted at 300 *x g* for 6 mins at 4 °C, washed three times with 1X PBS for 10 mins each time, and permeabilized with 200 µL of 0.5% Triton X-100 in PBS for 10 mins on ice. Cells were then washed twice with 1X PBS, and rinsed twice with 200 µl of 2X saline-sodium citrate (SSC; Quality Biological, Inc) buffer for 10 mins each time. The permeabilized cells were hybridized with 4 μg Dig-labeled DNA probe containing 250 µg t-RNA (Invitrogen), and 25 µg each of Cot-1 DNA (Invitrogen) and salmon sperm DNA (Invitrogen). The probe mixture was prepared by combining the DNA probe with the t-RNA and carrier DNA, dried in a speed vacuum, resuspended in 100% formamide, denatured at 80°C for 10 mins and quick chilled on ice. Then, the probe mixture was added to hybridization buffer containing a final concentration of 1X SSC, 50% formamide (Sigma), and 2.5% dextran sulfate (Sigma). The cells were incubated with the probe mixture containing hybridization buffer (200 μl) overnight at 37°C in a humidified chamber, washed with PBS for 10 mins at room temperature, followed by PBS plus 3% BSA (200 µl) for 1 hr at room temperature with gentle agitation. Primary antibody (Sigma) against the V5 tag of Foxa1 was diluted with PBS plus 3% BSA and incubated with cells at 4°C overnight, followed by washing three times with PBS containing 0.5% Tween-20 for 10 mins each. Next, cells were incubated with Fluorescein-conjugated anti-Digoxigenin antibody (Roche) and Cy3-conjugated anti-rabbit IgG secondary antibody (Jackson ImmunoResearch Laboratories) for 1 hr. Finally, the cells were washed two times each with PBS, and resuspended in 60 µl PBS for FISH analysis using the ImageStream cell analysis system. The nuclear stain DRAQ5 (ENZO Life Sciences) was added 5 mins prior to loading of cells into the ImageStream flow cell.

### Automated image acquisition and FISH analysis

Cells in suspension were automatically imaged in flow using the ImageStream 100 imaging cytometer (Amnis, now part of EMD Millipore, a division of Merck KGa, Darmstat, Germany). The ImageStream 100 acquires and processes six digital 10-bit multi-spectral images per cell at rates of up to 100 events per second. The images are captured simultaneously in six channels corresponding to brightfield, side scatter, and various emission spectra at a resolution of 0.5 µm per pixel. The ImageStream 100 is equipped with a halogen light source to produce the brightfield image. An adjustable 200 mW 488 nm laser is used to excite the samples to generate fluorescence and side scatter emission. For these studies, 5 channels were used. Alexa Fluor 488 (A488) was used to detect the Ras RNA FISH probe which emitted in channel 3. Cy3 was used to detect Foxa1 which emitted in channel 4. DRAQ5 was used to label the nuclear DNA which emitted in channel 6. Brightfield emitted in channel 5 and side scatter in channel 1. Single color controls were run for A488, Cy3 and DRAQ5 to correct for spectral crosstalk within the multispectral experimental data. A compensation matrix was derived using the single color controls that are acquired under the identical experimental conditions as the raw experimental data to which it is applied. Application of the compensation matrix to the following linear algebraic operation yields compensated intensity values corrected for spectral overlap across channels: Compensated Intensity  =  Inverse (Compensation Matrix) x Uncompensated Intensity. Following these initial compensations, cellular images were automatically processed using the IDEAS™ analysis software to interrogate the RNA FISH spot detection and co-localization with Foxa1 immunofluorescence per cell nucleus. The nucleus of each cell was automatically identified by DRAQ5 positive fluorescence. An initial region of interest (ROI) or ‘mask’ of the nucleus was defined by the border of DRAQ5 fluorescence and the image background. An input segmentation mask characterizes the background (from the top 4 and bottom 4 rows of the image) using a set of intensity and texture features. It then computes the same set of features for blocks of the entire image. A deviation score is computed for each of these blocks with respect to the background and a threshold for masking is determined from the deviation score histogram by finding the first robust minima after the first peak which would correspond to the background pixels. Spot detection was performed by automatically defining a spot mask representative of the FISH foci for each A488-positive image. The spot mask uses a standard top-hat operation on the input segmentation mask to identify the spots. The output of the top-hat operation on the image results in individual peaks of the bright pixels. The lower limit of the RNA FISH signal detection (i.e. the spot mask) is based on a user-defined threshold setting for the spot: background ratio. The user-defined threshold is simply the height of these peaks in the top-hat image. The choice of threshold applies to all images in all treatment conditions for consistency. The spot mask was further restricted to the region within the nuclear mask to ensure that no cells with FISH signals outside the cell nucleus were included in the analysis. Because a gate is set on cells with at least 1 spot, the smallest FISH size represents the border of the threshold for discriminating cells with 0 versus 1 spot. Spot count was calculated by using the IDEAS™ algorithm *spot count*. Spot count was used to calculate the number of independent spot masks per image. FISH spot intensity was automatically calculated by applying the IDEAS™ algorithm *intensity* to the spot mask. The intensity algorithm calculates the sum of the pixel intensity units in a defined mask minus the average intensity in the background of the image. Thus, spot intensity represents the total background-subtracted pixel intensity in the spot mask. Spot size is calculated by applying the IDEAS™ algorithm *area* that calculates the spot mask area in µm^2^. The mask area is then divided by the spot count to get the mean spot size per cell in µm. Both the mean spot size (µm) and mean spot intensity (total pixel intensity units divided by the mask area) features of the FISH signal were used as indicators of MMTV transcriptional output in each cell. Co-localization between Foxa1 and the FISH signal was accomplished using the IDEAS™ algorithm *bright detail similarity (BDS).* Specifically, the BDS R3 feature is designed to compare the similarity between two images and was used to quantify the co-localization of two probes in a defined region. Bright details of each image are first extracted using the top-hat morphological operator with a circular kernel of radius 3 (i.e. R3). Then, the Pearson's correlation coefficient of corresponding pixel pairs in the two bright detail images is computed. These values range between 0 (uncorrelated) and 1 (perfect correlation). To enhance the dynamic range of the coefficient values and to provide a more Gaussian-looking distribution, we log transform the coefficient so that it ranges from 0 to ∞.

### Quantitative reverse-transcriptase PCR (qRT-PCR)

Total RNA was extracted from 3134 cells using the RNeasy Mini Kit (Qiagen). The extracted RNA (1 µg) was used to prepare cDNA by reverse-transcription (RT) using the iScript cDNA Synthesis Kit (Bio Rad), followed by quantitative real-time PCR using the iQ SYBR Green Supermix (Bio-Rad) according to the manufacturer’s protocol. PCR reactions were performed at least three times in triplicate to analyze *Ras* and *β-Actin* mRNA expression and *Ras* mRNA levels were normalized to *β-Actin*. PCR primers were as follows: *Ras* Forward 5′-CTGACCATCCAGCTGATCCAG-3′; *Ras* Reverse 5′-ACACGTCTC CCCATCAATGAC-3′; *β-Actin* Forward 5′-TCCATCATGAAGT GTGACGT-3′; *β-Actin* Reverse 5′-TACTCCTGCTTGCTGATCCACAT-3′.

### Statistical analyses

Statistical analyses were performed using SigmaPlot 12.0 (Systat Software Inc., San Jose, CA). The Kolmogorov-Smirnov test revealed that the FISH intensity and size data did not follow a normal distribution (p< 0.001). Therefore, the degree of asymmetry within a cell sample was assessed using the skewness and kurtosis values reported by the Descriptive Statistics using SigmaPlot 12.0. Non-parametric ANOVAs (Kruskal-Wallis) on ranks were first used to determine whether any differences existed between all conditions at the p< 0.05 significance level. If significant differences were observed by the Kruskal-Wallis ANOVA, then the non-parametric Mann-Whitney U Test (a non-parametric post hoc test) was used to detect significant differences between individual pairs of samples. While the majority of data are shown using Box-and-whisker plots, a few figures were best represented as bar graphs displaying means + SEMs. For data within bar graphs, statistical significance was found to be similar using both parametric and non-parametric tests but the results from non-parametric tests were reported for consistency throughout the manuscript. The variability metric termed the % coefficient of variation [(std. dev/mean)*100%] was used to assess the volatility or spread within the FISH intensity and size distributions.

## Results

**Figure 1:**
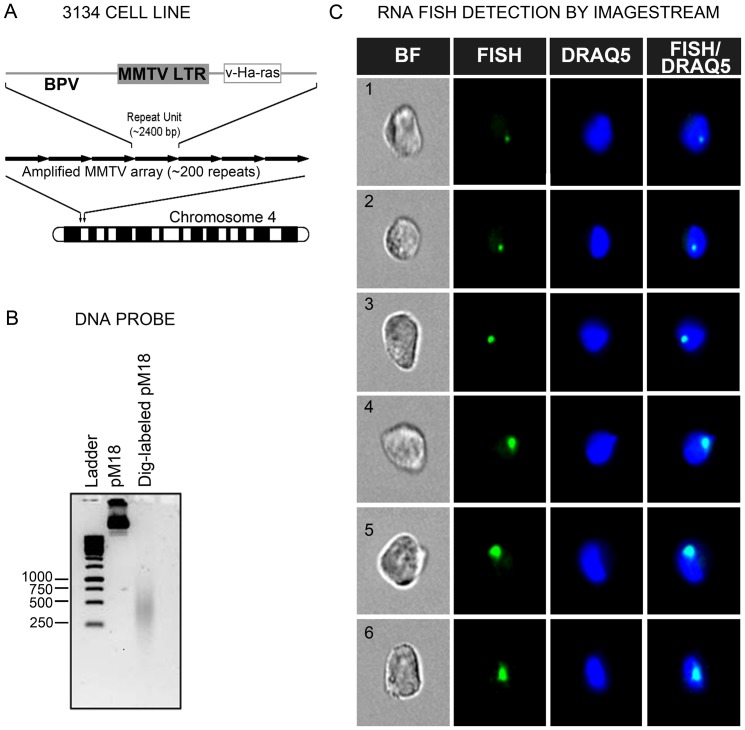
Automated detection of RNA FISH by imaging flow cytometry. (A) Organization of the MMTV-Ras transgene in the 3134 cell line. The 3134 cell line contains approximately 200 tandem repeats of MMTV-LTR driving viral Ras RNA on chromosome 4. (B) A Dig-labeled DNA probe was generated by nick translation of the pM18 plasmid containing the viral Ras coding sequence. Agarose gel electrophoresis revealed a DNA probe size within a 250–500 base pair range. (C) Imaging of cells following probe hybridization showed the presence of distinct nuclear FISH signals at a 40X magnification. The FISH signal was detected with anti-Dig secondary antibodies conjugated to a green-fluorescent dye. BF = Bright Field; DRAQ5 = nuclear stain.

**Figure 2:**
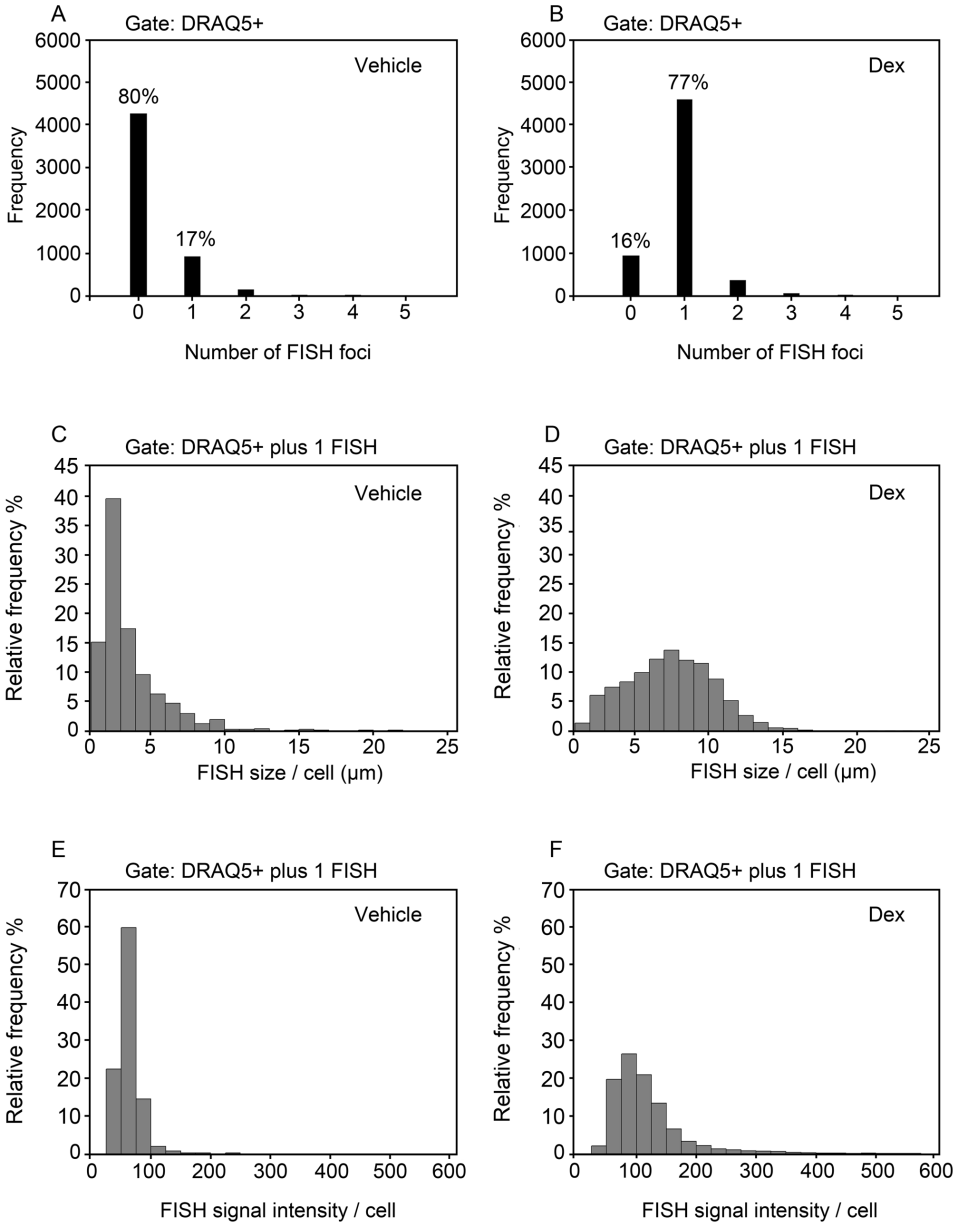
Hormone induction results in a heterogeneous MMTV-Ras transcriptional response in individual 3134 cells. (A, B) Frequency distribution of cells exhibiting 0, 1, or >1 FISH signals following vehicle or dexamethasone treatment for 1 hr. (C-F) Relative frequency distribution plots of the variability in the RNA FISH signal size (µm) (C, D) and FISH signal intensity (mean pixel intensity units) (E, F) detected in cells displaying 1 FISH signal following vehicle (n = 899) or dexamethasone (n = 4584) treatment.

RNA FISH was adapted from a standard protocol [21], with minor modifications to accommodate a cell suspension-based technique for analysis by flow-RNA FISH. The 3134 cell line used for RNA FISH studies harbors approximately 200 copies of an amplified MMTV LTR array driving a v-Ha-Ras reporter (Figure 1A). A DIG-labeled DNA probe was hybridized to Ras mRNA produced in response to MMTV promoter activation. The DNA probe was prepared by nick-translation of the pM18 plasmid containing the Ras reading frame and resulted in a 250–500 base-pair band smear of the probe (Figure 1B). Fluorescence imaging of hydrodynamically-focused cells in flow suspension following probe hybridization and antibody incubations showed the presence of distinct sub-spherical RNA foci colocalized with nuclear DRAQ5 at a 40X magnification (Figure 1C). Quantitative analysis of large cell samples (approximately 5,000 events) revealed major differences in the number of cells exhibiting FISH signals following vehicle and dexamethasone (dex) treatment for 1 hour. Approximately 80% of the 5,329 cells analyzed in the absence of hormone showed no FISH signals (Figure 2A), and 17% of the remaining cells displayed a small but detectable nuclear fluorescent spot indicative of basal MMTV transcription (Figure 2A). By contrast, only 16% of 5,936 cells analyzed following dex treatment were devoid of FISH signals (Figure 2B). The large majority of these dex-treated cells (i.e. 77%) displayed a single RNA FISH signal (Figure 2B). Standard microscopy studies of a 3134 cell line subclone previously noted that 10% of cells show FISH signals before hormone treatment, but 90% show FISH signals following 100 nM dex treatment for 1 hr [22]. Thus, our data are consistent with the earlier report of a dex-induced increase in the number of cells displaying RNA FISH signals. The present findings serve as ‘proof of concept’ that ImageStream may be used to detect hormone-dependent transcription within individual cells by flow-RNA FISH.

A small subset of cells displayed greater than one FISH signal in the vehicle- (3%) and dex-treated (7%) cell samples by ImageStream (Figure 2A-B). Other studies using DNA FISH of interphase nuclei have observed the presence of multiple arrays within a few cells, suggesting that gene duplication events could give rise to additional copies of the array [22]. For this reason, a gate was placed on the cell population to restrict analysis to only those cells displaying a single FISH signal. Analysis of the RNA FISH size per cell revealed a highly variable MMTV transcriptional response within individual cells. A relative frequency distribution plot of the RNA FISH size showed that more than 50% of the FISH-positive cells within the vehicle condition contain small FISH signals approaching the lower limit of detection within the range of 1.25 and 3.75 µm in diameter (Figure 2C). Furthermore, the relative frequency distribution plot revealed a positive skewed (i.e. longer right tail) profile with skewness and kurtosis (i.e. peakness) values of 2.779 and 12.066, respectively (Figure 2C). Dex treatment for 1 hr dramatically expanded the FISH size range between 1.25 to 15 µm per cell, and resulted in a relative frequency distribution profile with skewness and kurtosis values of 0.189 and –0.122, respectively (Figure 2D). Quantification of the RNA FISH signal intensity as another measure of MMTV transcription showed extensive cell-to-cell variability similar to that of the FISH size. A relative frequency distribution plot of the FISH signal intensity showed that most (i.e. 80%) of FISH-positive cells within the vehicle condition contain FISH signals within the range of 25 to 75 intensity units per cell (Figure 2E). This resulted in a positive skewed profile with skewness and kurtosis values of 3.004 and 18.876, respectively (Figure 2E). Dex treatment for 1 hr considerably expanded the FISH signal intensity range from 25 to 300 intensity units per cell, and resulted in a relative frequency distribution profile with skewness and kurtosis values of 3.004 and 13.807, respectively (Figure 2F). The large heterogeneous response observed for the RNA FISH size and intensity by analysis of over 4,500 individual cells following hormone treatment indicates probabilistic or stochastic mechanisms controlling MMTV transcription. Live cell imaging as well as *in vitro* biochemical studies have previously demonstrated rapid and dynamic interactions of GR and coregulators with the MMTV gene [16], [23], [24]. Probabilistic interactions among these and other regulatory factors with the MMTV promoter array could contribute to the heterogeneous transcriptional response characterized by a non-normal distribution in the RNA FISH size and intensity as observed in the present studies.

**Figure 3:**
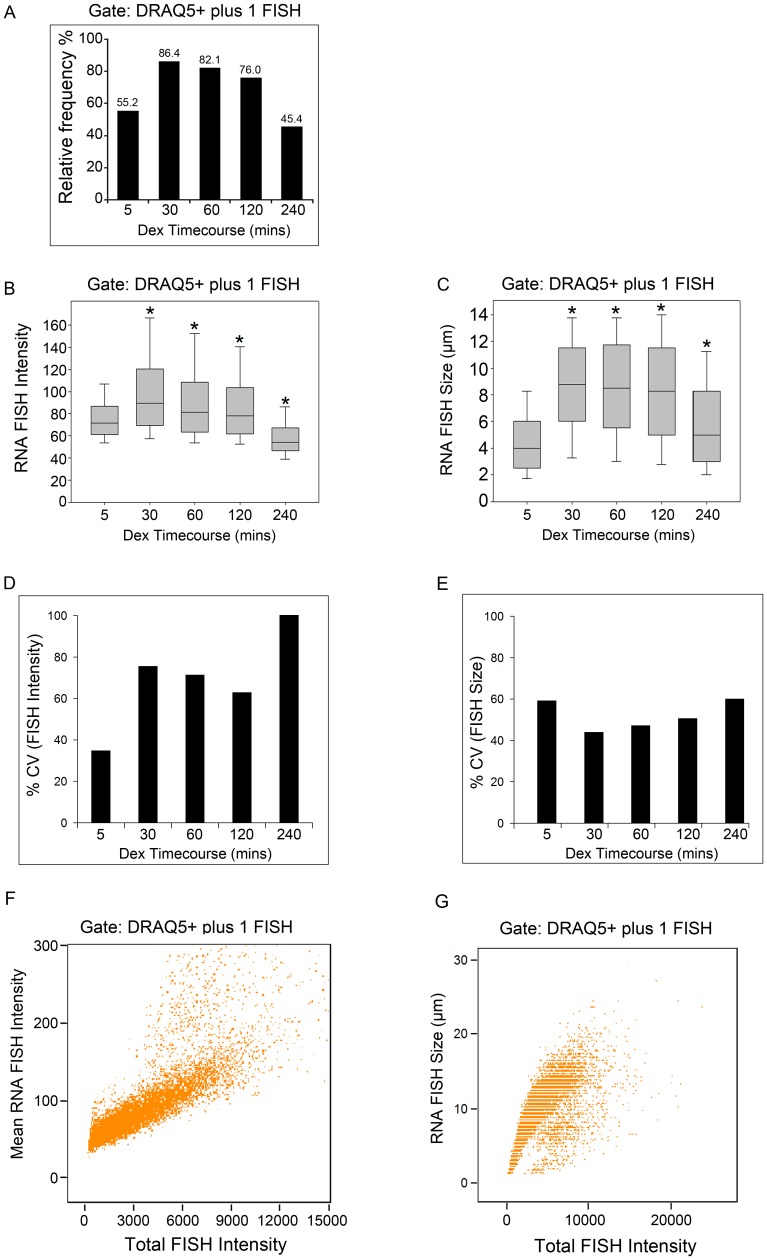
Time course experiments of the MMTV-Ras transcriptional response. (A) Frequency distribution of the percentage of cells exhibiting 1 FISH signal following dexamethasone treatment for 5, 30, 60, 120 and 240 mins. (B, C) Box-and-whisker plots of the RNA FISH signal intensity (mean pixel intensity units) and size (µm) distributions at each time point. (D, E) Computations of the individual cell-to-cell variability in the FISH signal intensity and size using the % coefficient of variation (% CV). Sample sizes for each time point are as follows: 5 mins (n = 7777), 30 mins (n = 10178), 60 mins (n = 11058), 120 mins (n = 10887) and 240 mins (n = 227). (F) Scatter plot showing a positive linear relationship between the FISH intensity (mean pixel intensity units) and the total FISH intensity (total pixel intensity units) within each FISH foci using the 60 min time point data (n = 11058). (G) Scatter plot showing a positive linear relationship between the FISH size (µm) and the total RNA FISH intensity (total pixel intensity units) using the 60 min time point data (n = 11058). * in (B,C), indicates a significant difference from the 5 mins time point as determined by the Mann-Whitney U Test (p< 0.001).

To evaluate how the variability in the FISH signal changes over time, time course experiments were performed following dex treatment for 5, 30, 60, 120 and 240 mins (Figure 3A). Approximately 86% of cells were positive for a single FISH signal at 30 mins following dex treatment, and the percentage of FISH-positive cells decreased steadily such that less than 50% of cells continued to show FISH signals at 240 mins following dex treatment (Figure 3A). Box-and-whisker plots were used to display the spread in the FISH intensity and size variability at the different time points. The FISH intensity and size changed significantly over time as determined by the Kruskal-Wallis ANOVA (p< 0.001) (Figure 3B-C). Then, differences in the FISH intensity and size at specific time points compared to that of 5 mins were evaluated using the Mann-Whitney U test. The median FISH intensity significantly increased from 71.5 intensity units at 5 mins to 89.7 intensity units at 30 mins, remained significantly higher at 60 and 120 mins, and decreased to 54.2 intensity units at 240 mins (p< 0.001) (Figure 3B). The median FISH size more than doubled from 4.00 µm at 5 mins to 8.75 µm at 30 mins of dex treatment (p< 0.001) (Figure 3C). The median FISH size also remained higher at all subsequent time points, including at 240 mins (median FISH size 5.00 µm) compared to that of the 5 mins time point (median FISH size 4.00 µm, p< 0.001) (Figure 3C). Next, the individual cell variability in the FISH intensity and size was calculated using the variability metric referred to as the % coefficient of variation (% CV) which represents the ratio of the standard deviation to the mean. We noted that the variability in the FISH intensity was more greatly affected by the duration of dex treatment than the FISH size (Figure 3D-E). These time course differences in the % CV for mean FISH intensity versus mean FISH size were observed despite the fact that mean FISH intensity and size values are both computed from the total FISH intensity, and are positively correlated with the total FISH intensity signal as expected (Figure 3F-G). Taken together, these time course studies suggest that the FISH intensity likely represents a more reliable readout of the variability in MMTV transcriptional responses in individual cells, even though population-wide changes in both the median FISH intensity and size are observed.

**Figure 4:**
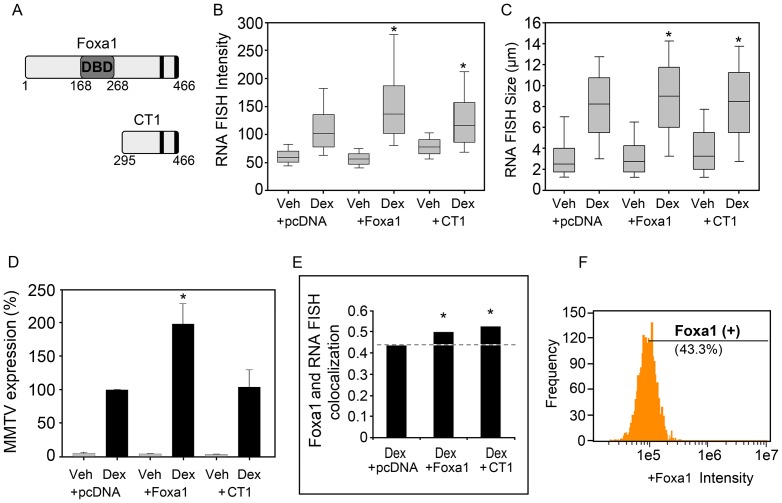
Foxa1 expression in the 3134 cell line enhances MMTV-Ras transcription. (A) Schematic of full-length Foxa1 and the C-terminal CT1 truncation mutant. The central DBD region interacts with DNA. Two C-terminal regions (black vertical bars) are important for core histone interactions. (B, C) Box-and-whisker plots of the RNA FISH signal intensity (mean pixel intensity units) and size (µm) distributions within each cell group. Sample sizes for each condition: Veh+pcDNA (n = 899), Dex+pcDNA (n = 4584), Veh+Foxa1 (n = 1067), Dex+Foxa1 (n = 2195), Veh+CT1 (n = 225), Dex+CT1 (n = 1959). (D) Comparative analysis of Ras RNA expression by qRT-PCR. Ras RNA expression is normalized to mouse β-actin. Data are expressed relative to that of Dex+pcDNA which is set to 100% (n≥3). (E) Bright Detail Similarity of Foxa1 colocalization with the RNA FISH foci. (F) Quantification of the percentage of total Foxa1-transfected cells showing positive Foxa1 positive (+) immunofluorescence. *, indicates a significant difference from the Dex+pcDNA group in each graph as determined by the Mann-Whitney U Test (p< 0.001).

To determine how chromatin accessibility influences gene expression, the pioneer factor Foxa1 was exogenously expressed in the 3134 cell line. The 3134 cell line lacks endogenous expression of Foxa1 and offers a good model system to quantify the pioneering function of Foxa1 on MMTV transcription. The unique chromatin remodeling function of Foxa1 and other members of the forkhead box family of transcription factors is related to their ability to target compact chromatin for remodeling so that additional factors may subsequently gain access to their respective DNA binding sites (for review, see [8]). Approximately 600 Foxa1 binding sites and 800–1200 GR binding sites are localized within the 200-copy tandem MMTV array. Either the full-length Foxa1 protein or a C-terminal (CT1) mutant was transfected in the 3134 cell line to evaluate Foxa1’s role in regulating GR-dependent MMTV transcription (Figure 4A). Each data set representative of the transfection (i.e. control pcDNA, Foxa1 or CT1) and treatment condition (Veh or Dex 1hr) was plotted as a box-and-whisker plot. Significant changes in the FISH intensity and size distributions were observed by the Kruskal-Wallis ANOVA (p< 0.001) (Figure 4B-C). Pairwise comparisons of FISH intensity and size across treatment and transfection conditions were carried out using the Mann-Whitney U test to first establish Foxa1’s pioneering effect prior to determining the impact on variability. Dex treatment of control pcDNA-transfected cells for 1 hr almost doubled the median FISH intensity from 59.5 to 102.1 intensity units (p< 0.001), and more than tripled the median FISH size from 2.5 µm to 8.25 µm (p< 0.001), indicative of a hormone-dependent increase in overall MMTV transcription (Figure 4B-C). To quantify Foxa1’s pioneering effect, the median FISH intensity and size changes were determined following Foxa1 transfection. Foxa1 further increased the FISH intensity relative to pcDNA, with a median increase from 102.1 intensity units observed with pcDNA to 136.2 intensity units observed with Foxa1 plus dex (p< 0.001) (Figure 4B). Unexpectedly, in the absence of dex, Foxa1 decreased the FISH signal intensity from 59.5 intensity units observed with pcDNA to 55.9 intensity units observed with Foxa1, suggesting that Foxa1 has a repressive effect on basal transcription (p< 0.001) (Figure 4B). Nevertheless, the increased transcriptional response seen by higher FISH intensity solely in the presence of dex is attributed to Foxa1 pioneering function to increase MMTV chromatin accessibility to ligand-activated GR (Figure 4B). The chromatin opening function of Foxa1 involves bimodal interactions with the DNA and core histone proteins. The CT1 mutant which lacks the N-terminus and central DNA binding domain (DBD) but preserves the C-terminal core histone binding region also resulted in a significant increase in the FISH intensity with a median change from 102.1 intensity units observed with pcDNA plus dex to 116.4 intensity units seen with CT1 plus dex (p< 0.001). However, the FISH intensity with Foxa1 was significantly higher than that observed with CT1 in the presence of dex (p< 0.001), indicating a sub-optimal transcriptional effect associated with the truncated protein relative to full-length Foxa1 (Figure 4B).

Changes in the RNA FISH size also support a Foxa1 pioneering role in GR-mediated MMTV transcription. Foxa1 significantly increased the FISH size compared to that of pcDNA in the presence of dex, with a median change in the FISH size from 8.25 to 9.0 µm (p< 0.001) (Figure 4C). No significant Foxa1-induced increase in the FISH size was observed in vehicle-treated cells (p =  0.117). Thus, Foxa1 pioneering function is observed by an increase in the median FISH size in the presence of dex. The CT1 mutant also increased the RNA FISH size compared to pcDNA in the presence of dex, with a median increase from 8.25 to 8.5 µm (p< 0.001) (Figure 4C). However, the FISH size with Foxa1 was significantly greater than that observed with CT1 in the presence of dex (p< 0.001) (Figure 4C). Parallel experiments were carried out to quantify MMTV-Ras mRNA expression by standard qRT-PCR analysis where the values obtained with Foxa1 plus dex were set to 100%. Significant differences across conditions were observed by the Kruskal-Wallis ANOVA (p< 0.001) (Figure 4D). qRT-PCR results were consistent with the RNA FISH signal intensity and size changes in that Foxa1 increased the Ras RNA levels compared to control pcDNA in the presence of dex as determined by the Mann-Whitney U Test (p< 0.001) (Figure 4D). However, the CT1 mutant in the presence of dex did not significantly increase Ras mRNA levels compared to control pcDNA plus dex (p =  0.641) (Figure 4D). To determine whether Foxa1 localizes to the RNA FISH foci, we used BDS analysis to compare the degree of Foxa1 fluorescence colocalized with the RNA FISH signal. The BDS value in the pcDNA plus dex condition was used as a baseline since no Foxa1 was expressed in these cells. Significant differences in Foxa1 colocalization with the FISH signal were observed by the Kruskal-Wallis ANOVA (p< 0.001) (Figure 4E). Foxa1 increased the correlation coefficient value to 0.50 compared to 0.43 observed with pcDNA as determined by the Mann-Whitney U Test (p< 0.001). The CT1 mutant also increased the correlation coefficient value to 0.53 from the 0.43 baseline observed with pcDNA, indicating increased colocalization for the Foxa1 mutant (p< 0.001) (Figure 4E). Next, we quantified the percentage of transfected cells showing nuclear Foxa1 immunofluorescence. Interestingly, this showed that only 43% of the Foxa1-transfected cells were positive for nuclear Foxa1 immunofluorescence (Figure 4F). Similarly, 44% of the CT1-transfected cells showed positive immunofluorescence for the CT1 Foxa1 mutant (data not shown). Since a significant Foxa1 effect was evident by RNA FISH and qRT-PCR despite only 43% of cells being positive for Foxa1 expression, additional gates were placed on the cells to further evaluate Foxa1 effects on the FISH intensity and size within Foxa1-enriched cells.

**Figure 5:**
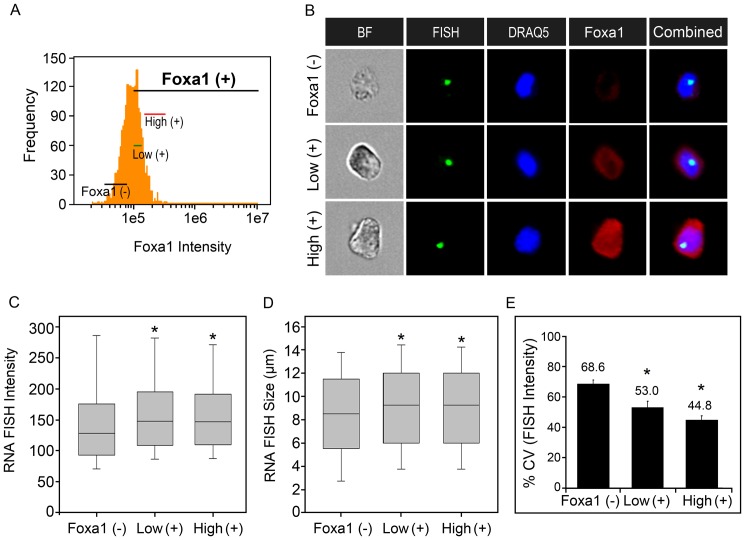
Foxa1 increases the median MMTV-Ras transcriptional response and decreases cell-to-cell variability in the FISH signal intensity. (A) Gating of sub-groups of cells representing no Foxa1 expression, low and high Foxa1 expression used for in-depth analyses of MMTV transcriptional variability. (B) A gallery of representative images of cells within the three sub-groups showing different degrees of Foxa1 immunofluorescence. (C, D) Box-and-whisker plots of the RNA FISH signal intensity (mean pixel intensity units) and size (µm) distributions within each sub-group. (E) Computation of the individual cell-to-cell variability in the FISH signal intensity using the % CV. Each % CV is calculated from greater than 100 cells. The % CV shown represents the mean + SEM from 4 independent experiments. *, indicates a significant difference from the Foxa1 negative (-) sub-group as determined by the Mann-Whitney U Test (p< 0.05).

Foxa1-transfected cells with a single RNA FISH foci were gated as Foxa1 negative (-) or Foxa1 positive (+) to provide a more precise measure of the MMTV transcriptional response rather than analysis of the entire cell population. In addition, the different levels of Foxa1 expression observed within the Foxa1 positive sub-population rendered it necessary to classify cells into sub-groups with closely matched levels of Foxa1 immunofluorescence (Figure 5A). Representative images of gated cells defined as Foxa1 (–), Low (+) and High (+) showed visible differences in the degree of nuclear Foxa1 expression (Figure 5B). Analysis of these gated cell sub-groups revealed significant differences in the FISH intensity (p< 0.001) and size (p< 0.015) distributions by the Kruskal-Wallis ANOVA (Figure 5C-D). Also, pairwise comparisons by the Mann-Whitney U Test showed specific changes in the FISH intensity and size related to Foxa1. Low Foxa1 levels significantly increased the RNA FISH signal intensity, with a median change from 127.8 intensity units in Foxa1-negative cells to 147.8 intensity units observed with low Foxa1 levels (p< 0.001) (Figure 5C). Similarly, high Foxa1 levels significantly increased the RNA FISH signal intensity, with a median change from 127.8 intensity units in Foxa1-negative cells to 146.5 intensity units observed with high Foxa1 (p< 0.001) (Figure 5C). There was no significant change in the FISH intensity between low and high Foxa1 by the Mann-Whitney U Test (p =  0.825) (Figure 5C). Pairwise comparisons of the RNA FISH size within the gated sub-groups were also performed. This showed that Foxa1 increased the FISH size with a median change from 8.5 µm observed in Foxa1-negative cells to 9.25 µm observed with either low Foxa1 (p< 0.015) or high Foxa1 (p< 0.015) (Figure 5D). Thus, both low and high Foxa1 produced similar pioneering effects related to increases in the FISH intensity and size.

Lastly, we determined whether individual cell differences in transcriptional variability are associated with the facilitative Foxa1 effect observed as an increase in median RNA FISH signal intensity and size following low and high Foxa1 expression. We computed the variability metric (% CV) as in Figure 3D-E. The mean % CV derived from four independent experiments showed significant differences in the FISH intensity among the three Foxa1 gated sub-groups by the Kruskal-Wallis ANOVA (p< 0.003) (Figure 5E). Pairwise comparisons by the Mann-Whitney U Test revealed a significant decrease in the % CV from 68.6% in Foxa1-negative cell to 53.0% with low Foxa1, indicating a reduction in the relative cell-to-cell variability in the FISH intensity (p< 0.03) (Figure 5E). High Foxa1 expression also significantly reduced the variability in the FISH intensity as indicated by a lower % CV value of 44.8% relative to that of Foxa1-negative cells (p< 0.03). There were no significant differences in % CV for FISH intensity between low and high Foxa1 (p =  0.2) (Figure 5E). The variability in the FISH size among the Foxa1-negative, low Foxa1 positive and high Foxa1 positive cells was also assessed. However, no significant differences in the % CV for FISH size were observed by the Kruskal-Wallis ANOVA (p =  0.815) (Figure S1). Another method for analysis of spread or variability surrounding the mean is referred to as the noise strength or Fano factor [1], [25], [26]. The Fano factor describes the population variability surrounding the average response and is calculated as the ratio between the variance and the mean. Analysis of the computed Fano factor values showed similar results to the % CV in that there were significant differences in the variability of the FISH intensity due to Foxa1 expression (p< 0.002), but not the FISH size (p =  0.48), by the Kruskal-Wallis ANOVA (Figure S2A-B). Specifically, there was a significant reduction in the variability in the FISH intensity due to low Foxa1 (p< 0.03) and high Foxa1 (p< 0.03) compared to the Foxa1 negative condition by pairwise comparisons of the mean Fano factor values using the Mann-Whitney U Test (Figure S2A). Therefore, the increase in the RNA FISH signal intensity along with the decrease in the variability suggests that Foxa1 could function to increase the fraction of time the MMTV promoter spends in an opened, accessible chromatin conformation relative to that of Foxa1-negative cells (Figure 5C and 5E). This Foxa1-induced effect favors an increased likelihood that the MMTV chromatin is accessible to GR and associated coregulators for promoter transactivation. As a consequence, there is an overall increase in transcriptional competence and at the same time there is a reduction in transcriptional variability between individual cells. These data therefore suggest that an additional function of the Foxa1 pioneer factor is to reduce transcriptional noise.

## Discussion

The intrinsic noise or variability in the transcriptional response observed within genetically-identical cells is related to several factors including random associations of regulatory factors and their co-occupancy at respective target genes, as well as fluctuations in the local chromatin environment at the regulatory sites. The amplified MMTV promoter array integrated at a single locus within the 3134 cell genome allows for direct visualization of regulatory factor interactions with chromatin. In response to the hormone dexamethasone, the GR, associated coregulators and members of the SWI/SNF family of chromatin remodeling complexes localize to the RNA FISH foci, suggesting targeting of these regulatory factors to the MMTV promoter [22], [27]. Moreover, live cell imaging studies of 3134 cell subclones expressing fluorescently tagged proteins have demonstrated a rapid and dynamic exchange of GR and coregulators with the MMTV array on a timescale of seconds [16], [23]. These dynamic interactions are captured as widely variable binding of regulatory factors to the MMTV chromatin within individually sampled cells that could contribute to a heterogeneous gene expression profile within the cell population [17], [18]. Heterogeneity in MMTV gene expression could also emanate from differences in the kinetics of factor loading. In the present study, we determined the extent of transcriptional variability by analyzing large numbers of cells following GR activation by dex treatment. We also determined the contribution of Foxa1-mediated chromatin remodeling to MMTV transcriptional variability following GR activation.

We show that dex activation of GR for 1 hr produces a broad shift in the population from a few cells displaying small FISH signals mostly under 5 µm to a large number of cells displaying widely variable FISH signals within the range of 1.25 to 15 µm in diameter (Figure 2A-D). A frequency distribution plot of the FISH size within the 17% of 5,329 cells exhibiting a FISH signal in the absence of hormone showed a highly skewed profile with a right tail distribution (Figure 2C). The shape of the distribution plot is reminiscent of the theoretical class II gene transition kinetics characterized by a slow transcriptional “On” state and fast “Off” state that yields a limited number of cells displaying low basal levels of transcription due to random chromatin opening for very short time intervals [25]. Activation of endogenous GR by dex treatment for 1 hr likely drove the RNA FISH size profile to the class III gene transition kinetics that features long “On” states and short “Off” states, thus giving rise to an increased number of cells displaying FISH signals (77% of the sampled cells treated with dex as opposed to 17% in the absence of hormone) and an expansion in the FISH size distribution (Figure 2B and 2D). Interestingly, the median RNA FISH size value observed for the entire cell population showed a deterministic increase in response to dex treatment even though the FISH size was variable at the individual cell level (compare 2^nd^ box-and-whisker in Figure 4C to Figure 2D). Similarly, the deterministic increase in the median RNA FISH intensity observed for the entire cell population was associated with highly variable changes at the individual cell level (compare 2^nd^ box-and-whisker in Figure 4B to Figure 2F).

To gain insight into how Foxa1-mediated chromatin remodeling influences gene expression, we first determined the median MMTV-Ras transcriptional response within the total cell population. The MMTV promoter harbors three Foxa1 DNA binding sites flanked by six glucocorticoid response elements (GREs) present within the nucleosomal B-C region of the promoter [14]. Exogenous Foxa1 localized to the FISH foci as quantified by BDS analysis clearly indicating that Foxa1 is targeted to the MMTV array (Figure 4E). A facilitative Foxa1 effect on GR-meditated MMTV-Ras expression was evident by a median increase in the RNA FISH signal intensity and size following dex treatment, and a repressive Foxa1 effect on basal transcription was observed in the FISH intensity in the absence of dex (Figure 4B-C). Biochemical experiments using *Xenopus* oocytes have shown that Foxa1 strongly enhances GR-induced MMTV transcription following corticosterone treatment, but Foxa1 binding to the MMTV promoter in the absence of hormone has little effect on MMTV transcription [28]. In our studies, a facilitative Foxa1 effect was also observed by qRT-PCR analysis of Ras expression relative to that of Foxa1 negative (i.e. pcDNA-transfected) cells in the presence of dex (Figure 4D). Therefore, the present RNA FISH and qRT-PCR studies are consistent with a Foxa1-mediated increase in transcriptional competence. A C-terminal mutant of Foxa1 that lacks the N-terminal half and central DNA binding domain (DBD) also localized to the FISH foci (Figure 4E), and increased the median RNA FISH intensity and size, although to a lesser degree than full-length Foxa1 (Figure 4B-C). The statistically significant effect is in part attributed to the high sensitivity of ImageStream and the ability to analyze large sample sizes as compared to qRT-PCR analysis which did not yield a statistically significant effect for the truncated Foxa1 mutant (Figure 4D). The mechanisms underlying the effect seen with the truncated Foxa1 mutant are unclear. The C-terminal region contributes to Foxa1’s pioneering function via interactions with core histones and also confers higher Foxa1 mobility in the nucleoplasm relative to linker histone H1 [11], [13]. It is conceivable that a non-specific affinity for chromatin via core histone interactions may allow the C-terminal Foxa1 mutant to marginally antagonize linker histone-mediated chromatin compaction and to promote chromatin de-compaction, thereby resulting in minor increases in the FISH signal intensity and size (Figure 4B-C). By contrast, more stable binding of full-length Foxa1 to chromatin via dual interactions to the DNA and core histones may enable more efficient antagonism of linker histones, thus resulting in a maximal facilitative effect on GR-induced MMTV transcription as evidenced by RNA FISH and qRT-PCR (Figure 4B-D). The N-terminal region within the context of full-length Foxa1 has also been shown to play a role in facilitating GR-mediated MMTV transcription [29]. Therefore, a combination of enhanced chromatin accessibility to the GR transcriptional complex and linker histone antagonism likely underlies Foxa1 pioneering function at the MMTV promoter.

How Foxa1 pioneering function impacts transcriptional variability has not been previously reported. Chromatin remodeling could generate stochastic gene expression by influencing promoter transitions between transcription factor accessible and inaccessible states [7]. We asked whether the Foxa1-mediated pioneering function influences stochastic gene expression by considering the individual cell-to-cell transcriptional variability as measured by the % CV and Fano factor. Our analysis focused on specific gated sub-populations of Foxa1-transfected cells. We reasoned that analysis of the total cell population would mask subtle Foxa1 effects associated with heterogeneous levels of Foxa1 expression as shown in Figure 5B. Additionally, the observation that roughly 50% of cells remained Foxa1-negative following transfection disfavored comparisons of the entire Foxa1-transfected cell population to the control pcDNA-transfected cell population as in Figure 4B-C. Comparisons of the FISH intensity and size within the Foxa1 negative, low Foxa1-positive and high Foxa1-positive cell sub-groups confirmed a pioneering role for Foxa1 in increasing GR-mediated MMTV transcription (Figure 5C-D). It has been noted elsewhere that the RNA FISH signal intensity may represent a more suitable indicator of transcription output than RNA FISH size because the total intensity signal is directly proportional to the number of RNA copies present within the RNA FISH foci [30]. However, in our studies both the RNA FISH size and intensity served as good predictors of Foxa1’s ability to increase GR-mediated MMTV transcriptional output (Figure 5C-D). By contrast, with regard to assessments of transcriptional stochasticity, only the FISH intensity served as a reliable indicator of variability in the MMTV transcriptional response (compare Figure 5E to Figure S1). Both low and high Foxa1 levels decreased the relative cell-to-cell variability in the FISH intensity as determined by lower % CV values indicative of decreased stochasticity (Figure 5E). Similarly, low and high Foxa1 decreased the variability in the FISH intensity, but not the FISH size, by assessments of Fano factor as another measure of variability (Figure S2A-B). Taken together, these results provide evidence for a novel function of Foxa1 to reduce transcriptional noise.

This noise reduction is most likely related to Foxa1’s pioneering function. Biochemical experiments have shown that MMTV chromatin in the 3134 cell line exists in multiple transitional states defined by different occupancy levels of linker histone H1 and other regulatory factors following dex treatment [31]. Therefore, the decrease in transcriptional variability (Figure 5E) and higher median transcriptional output (Figure 5C) in the FISH intensity observed following exogenous Foxa1 expression imply that Foxa1 chromatin remodeling increases the duration of time the MMTV promoter spends in an accessible state amenable to GR- and coregulator-mediated transactivation. Indeed, lower variability (as assessed by the Fano factor) and increased transcriptional output have been used to support promoter transition kinetics featuring longer “On” states and shorter “Off” states [25]. Future studies should consider the roles of other remodeling factors in regulating both transcriptional output and variability since GR coordinates the recruitment of multiple classes of chromatin remodelers to activate transcription. There is some evidence that cellular overexpression of components of the SWI/SNF family of chromatin remodeling factors or the p160 transcriptional coactivators increases the mean transcriptional output and decrease stochasticity as well [17]. By contrast, deletion of SWI/SNF factors has been shown to increase stochasticity [26]. Any comprehensive model of transcriptional regulation will require determinations of both cooperative and combinatorial interactions among different classes of chromatin remodelers, and also how they integrate at the level of a target gene to dynamically affect chromatin accessibility and transcriptional output.

In conclusion, the present studies demonstrate how a snapshot of gene expression within a large cell population by flow-RNA FISH may provide insights into stochastic gene expression. Quantification of the RNA FISH size and intensity revealed the extent of biological heterogeneity that exists within the clonal 3134 cell population. We show that exogenous overexpression of the pioneer factor Foxa1 decreases the transcriptional variability or noise possibly by driving chromatin transitions from an inaccessible to a more opened and transcriptionally-competent state. These results suggest that Foxa1 pioneering function serves dual roles in increasing transcriptional competence and decreasing noise.

## Supporting Information

Figure S1 **Foxa1 does not affect the variability in the FISH size.** Computation of the individual cell-to-cell variability in the FISH size using the % CV. Each % CV is calculated from the same source data used for determination of the % CV for FISH intensity in Figure 5E. NS, indicates not statistically significant from the Foxa1 negative (-) sub-group as determined by the Mann-Whitney U Test.
(TIF)

Figure S2 **Foxa1 decreases variability in the FISH intensity but not the FISH size as determined by a different noise metric termed the Fano factor.** (A, B) Computations of the individual cell-to-cell variability in the FISH signal intensity and size by Fano factor calculation (variance/mean). The Fano factor is calculated from the identical data used for determination of the % CV for the FISH intensity (Figure 5E) and size (Figure S1). Error bars represent +SEM; *, indicates a significant difference from the Foxa1 negative (-) sub-group as determined by the Mann-Whitney U Test (p<0.05). NS, indicates not significant from the Foxa1 negative (-) sub-group.
(TIF)

## References

[1] Kaern M, Elston TC, Blake WJ, Collins JJ (2005) Stochasticity in gene expression: from theories to phenotypes. Nat Rev Genet 6: 451–464. 15883588 10.1038/nrg1615

[2] Raj A, van Oudenaarden A (2009) Single-molecule approaches to stochastic gene expression. Annu Rev Biophys 38: 255–270. 19416069 10.1146/annurev.biophys.37.032807.125928 PMC3126657

[3] Bengtsson M, Stahlberg A, Rorsman P, Kubista M (2005) Gene expression profiling in single cells from the pancreatic islets of Langerhans reveals lognormal distribution of mRNA levels. Genome Res 15: 1388–1392. 16204192 10.1101/gr.3820805 PMC1240081

[4] Raj A, van Oudenaarden A (2008) Nature, nurture, or chance: stochastic gene expression and its consequences. Cell 135: 216–226. 18957198 10.1016/j.cell.2008.09.050 PMC3118044

[5] Gandhi SJ, Zenklusen D, Lionnet T, Singer RH (2011) Transcription of functionally related constitutive genes is not coordinated. Nat Struct Mol Biol 18: 27–34. 21131977 10.1038/nsmb.1934 PMC3058351

[6] Zenklusen D, Larson DR, Singer RH (2008) Single-RNA counting reveals alternative modes of gene expression in yeast. Nat Struct Mol Biol 15: 1263–1271. 19011635 10.1038/nsmb.1514 PMC3154325

[7] Raj A, Peskin CS, Tranchina D, Vargas DY, Tyagi S (2006) Stochastic mRNA synthesis in mammalian cells. PLoS Biol 4: e309. 17048983 10.1371/journal.pbio.0040309 PMC1563489

[8] Lalmansingh AS, Karmakar S, Jin Y, Nagaich AK (2012) Multiple modes of chromatin remodeling by Forkhead box proteins. Biochim Biophys Acta 1819: 707–715. 22406422 10.1016/j.bbagrm.2012.02.018

[9] Zaret KS, Carroll JS (2011) Pioneer transcription factors: establishing competence for gene expression. Genes Dev 25: 2227–2241. 22056668 10.1101/gad.176826.111 PMC3219227

[10] Clark KL, Halay ED, Lai E, Burley SK (1993) Co-crystal structure of the HNF-3/fork head DNA-recognition motif resembles histone H5. Nature 364: 412–420. 8332212 10.1038/364412a0

[11] Cirillo LA, Lin FR, Cuesta I, Friedman D, Jarnik M, et al (2002) Opening of compacted chromatin by early developmental transcription factors HNF3 (FoxA) and GATA-4. Mol Cell 9: 279–289. 11864602 10.1016/s1097-2765(02)00459-8

[12] Pani L, Overdier DG, Porcella A, Qian X, Lai E, et al (1992) Hepatocyte nuclear factor 3 beta contains two transcriptional activation domains, one of which is novel and conserved with the Drosophila fork head protein. Mol Cell Biol 12: 3723–3732. 1324404 10.1128/mcb.12.9.3723-3732.1992 PMC360231

[13] Zaret KS, Caravaca JM, Tulin A, Sekiya T (2010) Nuclear mobility and mitotic chromosome binding: similarities between pioneer transcription factor FoxA and linker histone H1. Cold Spring Harb Symp Quant Biol 75: 219–226. 21502411 10.1101/sqb.2010.75.061

[14] Holmqvist PH, Belikov S, Zaret KS, Wrange O (2005) FoxA1 binding to the MMTV LTR modulates chromatin structure and transcription. Exp Cell Res 304: 593–603. 15748903 10.1016/j.yexcr.2004.12.002

[15] Kramer PR, Fragoso G, Pennie W, Htun H, Hager GL, et al (1999) Transcriptional state of the mouse mammary tumor virus promoter can affect topological domain size in vivo. J Biol Chem 274: 28590–28597. 10497225 10.1074/jbc.274.40.28590

[16] McNally JG, Muller WG, Walker D, Wolford R, Hager GL (2000) The glucocorticoid receptor: rapid exchange with regulatory sites in living cells. Science 287 1262–1265: 8277. 10.1126/science.287.5456.1262 10678832

[17] Voss TC, Schiltz RL, Sung MH, Johnson TA, John S, et al (2009) Combinatorial probabilistic chromatin interactions produce transcriptional heterogeneity. J Cell Sci 122: 345–356. 19126674 10.1242/jcs.035865 PMC2724732

[18] Voss TC, John S, Hager GL (2006) Single-cell analysis of glucocorticoid receptor action reveals that stochastic post-chromatin association mechanisms regulate ligand-specific transcription. Mol Endocrinol 20: 2641–2655. 16873444 10.1210/me.2006-0091

[19] Bresnick EH, John S, Berard DS, LeFebvre P, Hager GL (1990) Glucocorticoid receptor-dependent disruption of a specific nucleosome on the mouse mammary tumor virus promoter is prevented by sodium butyrate. Proc Natl Acad Sci U S A 87: 3977–3981. 2160080 10.1073/pnas.87.10.3977 PMC54027

[20] Ostrowski MC, Richard-Foy H, Wolford RG, Berard DS, Hager GL (1983) Glucocorticoid regulation of transcription at an amplified, episomal promoter. Mol Cell Biol 3: 2045–2057. 6318079 10.1128/mcb.3.11.2045-2057.1983 PMC370071

[21] Rayasam GV, Elbi C, Walker DA, Wolford R, Fletcher TM, et al (2005) Ligand-specific dynamics of the progesterone receptor in living cells and during chromatin remodeling in vitro. Mol Cell Biol 25: 2406–2418. 15743833 10.1128/MCB.25.6.2406-2418.2005 PMC1061598

[22] Muller WG, Walker D, Hager GL, McNally JG (2001) Large-scale chromatin decondensation and recondensation regulated by transcription from a natural promoter. J Cell Biol 154: 33–48. 11448988 10.1083/jcb.200011069 PMC2196867

[23] Becker M, Baumann C, John S, Walker DA, Vigneron M, et al (2002) Dynamic behavior of transcription factors on a natural promoter in living cells. EMBO Rep 3: 1188–1194. 12446572 10.1093/embo-reports/kvf244 PMC1308318

[24] Nagaich AK, Walker DA, Wolford R, Hager GL (2004) Rapid periodic binding and displacement of the glucocorticoid receptor during chromatin remodeling. Mol Cell 14: 163–174. 15099516 10.1016/s1097-2765(04)00178-9

[25] Munsky B, Neuert G, van Oudenaarden A (2012) Using gene expression noise to understand gene regulation. Science 336: 183–187. 22499939 10.1126/science.1216379 PMC3358231

[26] Raser JM, O'Shea EK (2004) Control of stochasticity in eukaryotic gene expression. Science 304: 1811–1814. 15166317 10.1126/science.1098641 PMC1410811

[27] Johnson TA, Elbi C, Parekh BS, Hager GL, John S (2008) Chromatin remodeling complexes interact dynamically with a glucocorticoid receptor-regulated promoter. Mol Biol Cell 19: 3308–3322. 18508913 10.1091/mbc.E08-02-0123 PMC2488306

[28] Belikov S, Astrand C, Wrange O (2009) FoxA1 binding directs chromatin structure and the functional response of a glucocorticoid receptor-regulated promoter. Mol Cell Biol 29: 5413–5425. 19687299 10.1128/MCB.00368-09 PMC2756877

[29] Belikov S, Holmqvist PH, Astrand C, Wrange O (2012) FoxA1 and glucocorticoid receptor crosstalk via histone H4K16 acetylation at a hormone regulated enhancer. Exp Cell Res 318: 61–74. 22001115 10.1016/j.yexcr.2011.09.016

[30] Femino AM, Fay FS, Fogarty K, Singer RH (1998) Visualization of single RNA transcripts in situ. Science 280: 585–590. 9554849 10.1126/science.280.5363.585

[31] Georgel PT, Fletcher TM, Hager GL, Hansen JC (2003) Formation of higher-order secondary and tertiary chromatin structures by genomic mouse mammary tumor virus promoters. Genes Dev 17: 1617–1629. 12842912 10.1101/gad.1097603 PMC196134

