# Supplementary figures and images for: High-Throughput RNA FISH Analysis by Imaging Flow Cytometry Reveals That Pioneer Factor Foxa1 Reduces Transcriptional Stochasticity
Source: PLoS One. 2013 Sep 20;8(9):e76043. doi: 10.1371/journal.pone.0076043 (PMC3779185; doi:10.1371/journal.pone.0076043)

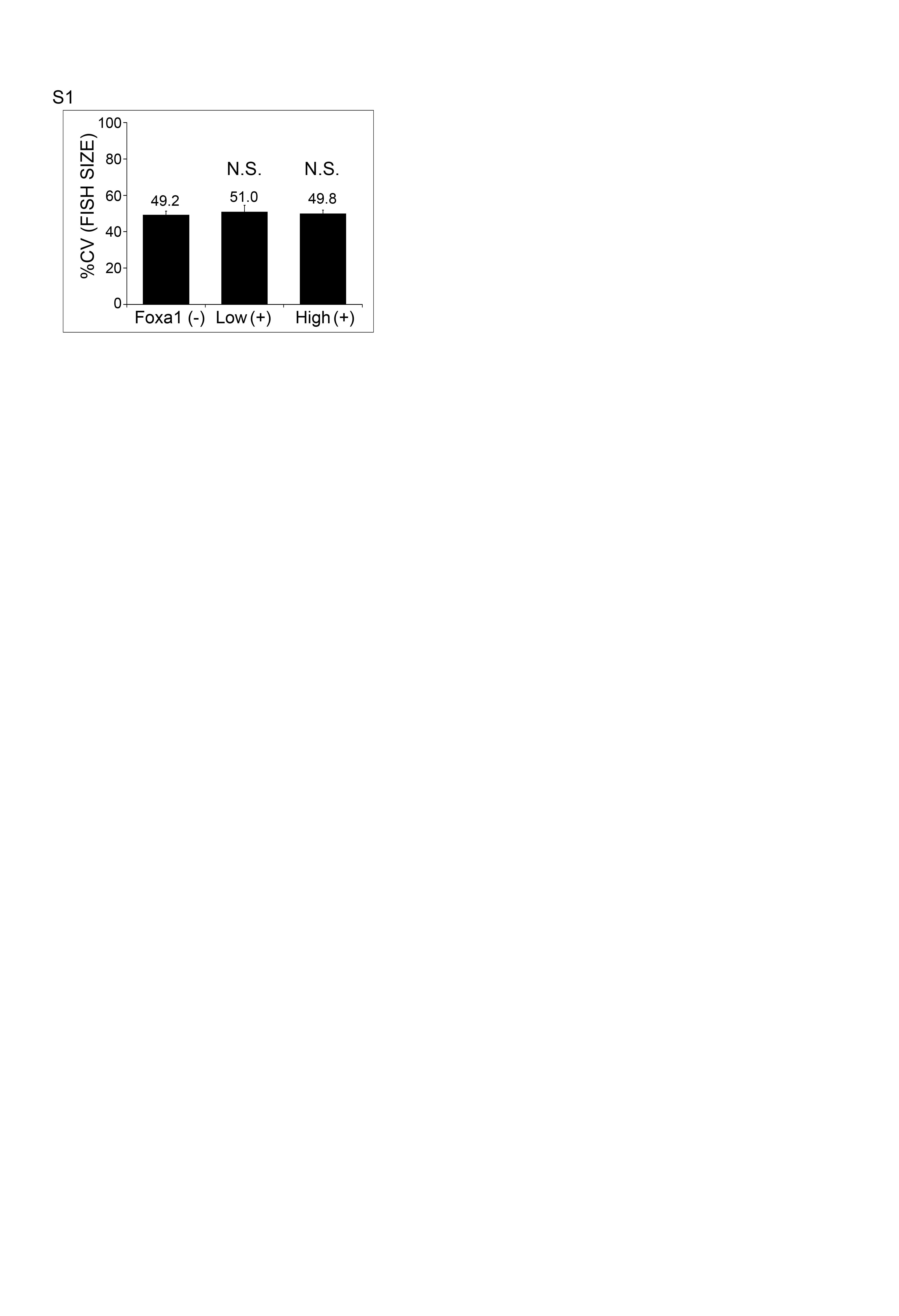

Supplement: Figure S1 — Foxa1 does not affect the variability in the FISH size. Computation of the individual cell-to-cell variability in the FISH size using the % CV. Each % CV is calculated from the same source data used for determination of the % CV for FISH intensity in Figure 5E. NS, indicates not statistically significant from the Foxa1 negative (-) sub-group as determined by the Mann-Whitney U Test. (TIF) [file pone.0076043.s001.tif]

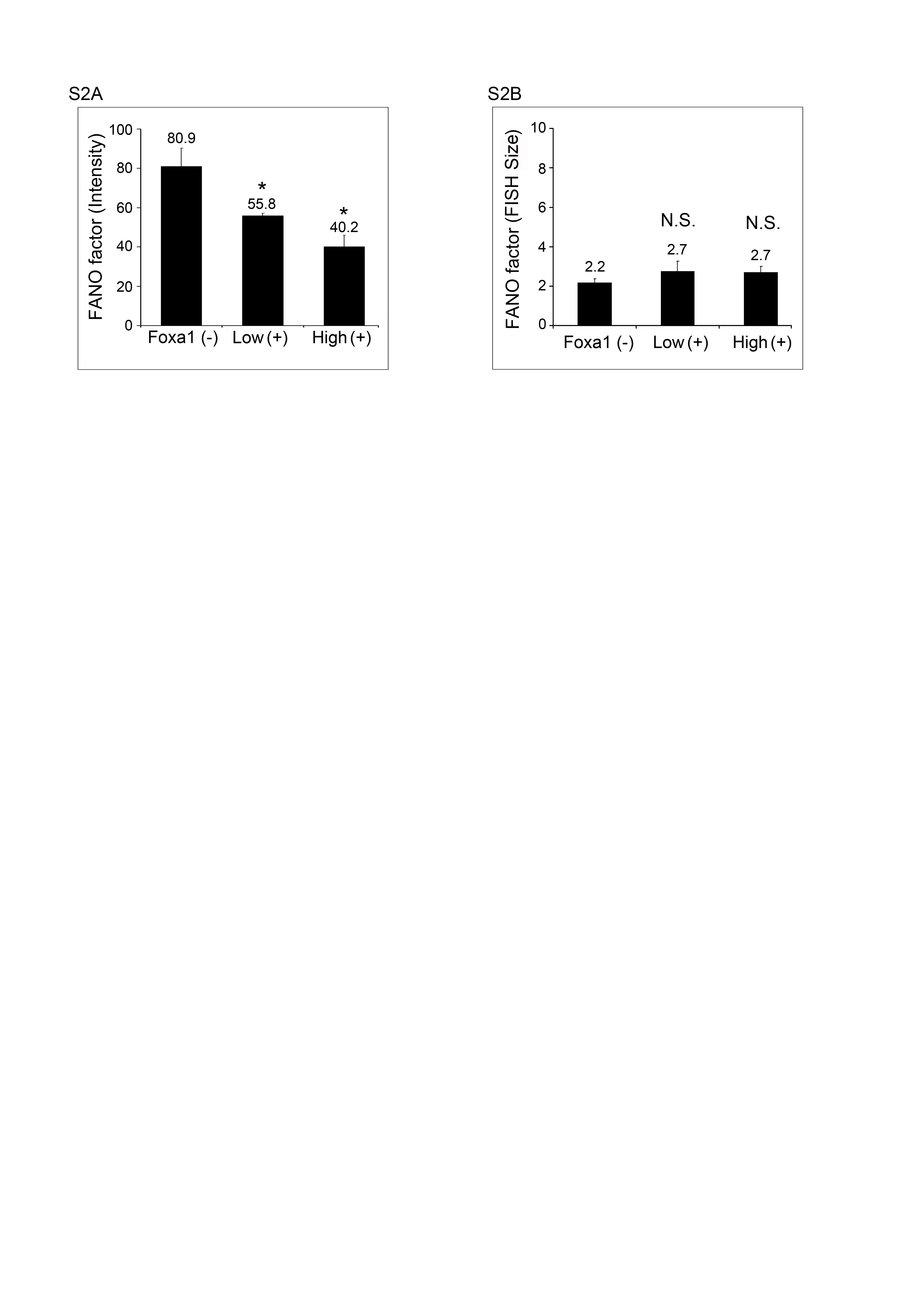

Supplement: Figure S2 — Foxa1 decreases variability in the FISH intensity but not the FISH size as determined by a different noise metric termed the Fano factor. (A, B) Computations of the individual cell-to-cell variability in the FISH signal intensity and size by Fano factor calculation (variance/mean). The Fano factor is calculated from the identical data used for determination of the % CV for the FISH intensity (Figure 5E) and size (Figure S1). Error bars represent +SEM; *, indicates a significant difference from the Foxa1 negative (-) sub-group as determined by the Mann-Whitney U Test (p<0.05). NS, indicates not significant from the Foxa1 negative (-) sub-group. (TIF) [file pone.0076043.s002.tif]
